# Competencies required when police patrol officers encounter people in suicidal crisis: a Delphi study

**DOI:** 10.1186/s40352-026-00442-z

**Published:** 2026-07-21

**Authors:** Martin Olsson, Henrik Andersson, Ola Kronkvist, Anders Svensson

**Affiliations:** 1 Department of Health and Caring Sciences, Linnaeus University, Vaxjo, Sweden; 2 Faculty of Caring Science, Work Life and Social Welfare, University of Borås, Borås, Sweden; 3 Centre of Interprofessional Cooperation within Emergency Care (CICE), Linnaeus University, Vaxjo, Sweden; 4 Department of Criminology and Policing, Linnaeus University, Vaxjo, Sweden

**Keywords:** Mental health, Police patrol officer, Suicidal crisis

## Abstract

**Introduction:**

Suicide is a leading cause of death globally and represents a common, resource-intensive duty for police patrol officers (PPOs). Responding to suicidal crises is often traumatic, and PPOs rarely receive comprehensive training, leaving them inadequately prepared. Police authorities frequently assume operational responsibility in such situations, encompassing emotional, legal, tactical and safety considerations.

**Aim:**

This study aimed to explore and describe the competencies required of PPOs when encountering individuals in suicidal crisis.

**Method:**

An exploratory, descriptive design was employed using a modified Delphi technique. Descriptions provided by PPOs of encounters with individuals in suicidal crisis were used to inform the development of items for a two-round questionnaire distributed to an expert panel. Experts were defined as individuals employed by the Swedish Police Authority or a university college, with additional training and/or teaching experience related to managing suicidal crises.

**Results:**

Of the 50 experts invited, 43 completed both rounds, yielding a response rate of 86%. Consensus was achieved on 50 items, categorised into knowledge (*n* = 14), skills (*n* = 22) and attitudes (*n* = 14).

**Conclusion:**

The findings highlight the wide-ranging competencies required of PPOs, including policing-specific skills related to safety, interpersonal communication and psychiatric knowledge such as understanding mental disorders. Attitudinal competencies, such as demonstrating compassion and adopting a problem-solving approach, were also emphasised.

## Introduction

Police patrol officers (PPOs) face a variety of challenges that require them to adapt to a wide range of encounters, drawing on a combination of knowledge, skills and attitudes (Polismyndigheten 2021, Justitiedepartementet [Ministry of Justice] 2016). One of the most challenging situations is responding to ongoing attempts or serious threats of imminent suicide (Chidgey et al. 2019). Hereafter, this will be referred to as a suicidal crisis. Suicide is one of the leading causes of death worldwide, claiming more than 700,000 lives each year (World Health Organization 2021). In Sweden, about 1,500 people die by suicide every year, and 11,000 people received care at a hospital for suicide attempts or other intentional self-harm (Folkhälsomyndigheten 2021).

A suicidal crisis is often preceded by struggles with thoughts, fantasies or considerations about ending one’s life, that is, suicidal ideation. Suicidal ideation is usually understood to arise from a combination of unbearable psychological pain and hopelessness. In addition, a number of well-established risk factors are associated with increased suicide risk, including previous suicide attempts, substance misuse, mental disorders, and exposure to adverse life events. These factors often interact, with recent stressors and the accumulation of multiple risks further increasing vulnerability (Favril et al. 2022). However, suicidal ideation alone does not fully provide a framework for understanding the transition to a suicidal crisis (Klonsky et al. 2021). Reported rates of suicidal ideation leading to an actual suicide attempt vary significantly between studies, with estimates ranging from 2.6% to 37% (Haregu et al. 2023). The transition from strong suicidal ideation to a suicide attempt is facilitated by dispositional, acquired and practical factors. Suicidal capability primarily involves overcoming fear of attempting suicide and having the means and knowledge to act, such as access to lethal means (Klonsky et al. 2021). Personality traits such as neuroticism have been identified as potential risk factors. Furthermore, deficits in emotion regulation, particularly difficulties in managing negative affect such as anxiety or sadness, may increase vulnerability during adolescence, a developmental stage in which suicide ranks among the leading causes of death (Planellas and Calderón 2022, Colmenero-Navarrete et al. 2022).

The involvement of PPOs in handling mental health emergencies, including responding to suicidal crises, is globally considered resource-intensive (Dempsey et al. 2020). There is a lack of reliable statistics tracking the exact extent to which the Swedish police respond to suicidal crises. This also reflects difficulties in capturing the circumstances of such incidents, including distinctions between mental health emergencies, suicidal ideation, and suicidal crisis, as well as the absence of data on how these cases are resourced. Nevertheless, available reports suggest an upward trend in the use of police resources for such cases, with these incidents constituting a substantial and increasing proportion of overall police duties (Polismyndigheten 2022, Socialdepartementet 2025). Such incidents are often characterised by urgency, uncertainty, and potential safety risks, placing considerable demands on responding PPOs (Steele et al. 2024). In the Swedish context, this involvement is partly grounded in the police’s legal authority to undertake compulsory transport to healthcare facilities for psychiatric evaluation under the Compulsory Psychiatric Care Act, and partly reflects the police service’s broader societal mandate to maintain public safety (Lag 1991, Polislagen 1984). Exercising this authority requires a legally informed assessment of whether the criteria for compulsory conveyance are met, underscoring the need for PPOs to possess adequate legal competencies. This responsibility of PPOs becomes particularly pronounced in encounters where the presence of firearms, knives or other weapons necessitates immediate intervention due to safety concerns. Although the use of knives or other sharp objects is commonly associated with self-harming behaviour, it is not always straightforward to distinguish such acts from a suicidal crisis (Beckman et al. 2018, Statens offentliga utredningar 2024). In Sweden, the most common methods of suicide are hanging and poisoning. However, the methods differ between men and women, and the third most common method for men is the use of firearms (Folkhälsomyndigheten 2024). Competencies traditionally associated with PPOs, such as tactical procedures and negotiation skills, are essential for securing the scene and resolving the situation (Hansson et al. 2021). This underscores the difficulty of police work, where PPOs encounter situations that demand tactical decision-making but also a compassionate understanding of a person’s mental state and emotional distress. While the police authorities frequently assume operational responsibility in responding to people in suicidal crisis, interventions are often carried out in collaboration with Emergency Medical Services (EMS) and the Fire and Rescue Service (FRS) through co-response models. Such collaborative approaches necessitate an understanding of interprofessional collaboration (Blais and Brisebois 2021, Marcus and Stergiopoulos 2022). Previous research has demonstrated that police presence may have an escalating impact on individuals’ emotional responses, particularly among young people, where encounters can be experienced as distressing or anxiety-provoking. This may be of particular concern in the context of suicidal crises, where individuals are already highly vulnerable and sensitive to external stressors, potentially exacerbating the situation (Colmenero-Navarrete et al. 2022, Jackson et al. 2019). This also requires awareness that police presence may, in rare cases, be associated with situations in which individuals attempt to provoke a lethal response from police, sometimes described as “suicide by cop” (Jordan et al. 2020).

Responding to a suicidal crisis is considered potentially traumatic, and PPOs rarely receive extensive training, leaving them feeling inadequately prepared (Jordan et al. 2020, O’Reilly et al. 2025). There are several competencies relevant to managing suicidal crises that are generally associated with formal mental health training. These competencies may be grounded in medical and/or nursing sciences, such as knowledge of psychiatry, clinical reasoning skills, and a compassionate, person-centred approach (Colmenero-Navarrete et al. 2022). In Sweden, the Swedish Police Authority provides a national curriculum for the 2.5-year police education programme. This curriculum outlines a general educational framework but does not specify detailed competency requirements related to psychiatry or the management of people in suicidal crises (Polismyndigheten 2022). Consequently, each of the five university colleges offering the police education programme has the autonomy to structure their courses based on local syllabuses, which may result in variation in the extent and content of training related to management of suicidal crises. Various local and national in-service training programmes related to mental health and the management of suicidal crises are available for active-duty PPOs; however, these are generally brief and vary considerably in focus and depth. The competency profiles associated with these programmes also vary: some offer a broad introduction to mental health awareness, whereas others are specifically designed to enhance communication skills for managing suicidal crises (Osteen et al. 2021). A national suicide prevention strategy has identified the need for improved training on suicidal crises, including training for responding PPOs. The Swedish Police Authority is striving to strengthen PPOs’ competencies in managing mental health issues, including responding to suicidal crises (Socialdepartementet 2025). However, the variation in training provision and the lack of clearly defined competency requirements highlight the need to systematically identify the competencies required of PPOs in such encounters. Addressing this gap is critical not only for individual professional development but also for informing curriculum design and strengthening organisational preparedness. This should be understood in the context of reducing police violence, minimising coercion and improving responses to support wellbeing and recovery. Consequently, the competencies of Swedish PPOs in handling suicidal crises may vary and, in some cases, be insufficient.

To summarise, the response to a suicidal crisis is multi-faceted, shaped by the specific circumstances, the method involved, and the person’s mental state and needs. These are high-stakes encounters where responding PPOs play a critical role in influencing the outcome and potentially preventing serious injury or death. In addition to ensuring scene safety, PPOs can offer emotional support and, either voluntarily or through compulsory transport, facilitate a psychiatric evaluation. This underscores the importance of exploring the competencies required of PPOs when encountering people in suicidal crisis, as this may inform professional development, police education, and operational preparedness.

## Aim

The aim was to explore and describe police patrol officers’ competency requirements when encountering people in suicidal crisis.

## Method

### Design

An exploratory and descriptive design was adopted, using a modified Delphi technique. The Delphi technique is an iterative process that synthesises individual opinions to reach a consensus among a panel of experts (Keeney et al. 2011).

### Study setting

The study was conducted in Sweden, which is administratively divided into seven police regions. Each region holds responsibility for policing within its designated geographical area. Experts from all seven regions participated in the study.

### The experts

An expert is someone with knowledge and experience of the issue under investigation (i.e. a specialist in their field or an informed individual (Keeney et al. 2011)). In this study, an expert was defined as an individual (i) employed by the Swedish Police Authority or by a university college delivering police education programmes, and (ii) possessing additional training and/or teaching experience related to the management of suicidal crises. Additional training included being trained as crisis negotiators and/or as instructors in mental health first aid for suicidal crises. Teaching experience referred to instructors within the Police Authority or a university college who taught PPOs strategies for managing suicidal crises. Sampling was purposive to ensure that invited experts had this acquired expertise for the study. The experts were identified and selected by the authors in collaboration with a research group focusing on mental health in policing. From their network, an initial list of potential experts was crafted and later revised and modified by the authors. A group of 56 experts was agreed upon. They were contacted via email and received both written and verbal information about the study, the latter through a pre-recorded video. An additional twelve individuals who met the inclusion criteria were identified through referrals provided by those initially contacted and were subsequently invited to participate. Of the 68 invited, 50 (*n* = 50) agreed to participate. Table 1 shows the demographic characteristics of the responding experts.

**Table 1:** Demographic characteristics of the responding experts

Characteristic	Round 1	Round 2
Experts (N)	47	43
Age (years), range and mean	26–62 (M = 43.1)	26–62 (M = 43.3)
Male (n)	28	27
Female (n)	19	16
Place of employment: Swedish Police Authority (n)	35	32
Place of employment: Police education (university college) (n)	12	11
Work experience (years), range and mean	1–40 (M = 13.9)	1–40 (M = 13.8)
Additional training in managing suicidal crises (n)	42	41
Teaching experience related to suicidal crises (n)	45	43

### Basis of the study

This study used a modified Delphi technique to generate items for inclusion in a two-round questionnaire distributed to a panel of experts. Initial competency items were derived from a secondary analysis of qualitative interview data originally collected in a prior study of Swedish PPOs’ encounters with people in suicidal crisis (Olsson et al. 2026). The dataset comprised 26 semi-structured interviews in which PPOs described 94 encounters, which were re-analysed to identify competencies relevant to such situations.

This provided a comprehensive description of the encounter, along with insights into the competencies reflected in the strategies and tactics employed. To extract described competencies, the first author carried out a manifest content analysis according to Erlingsson and Brysiewicz (2017) of the interviews. The initial coding and identification of competencies were conducted by the first author, who has a background in emergency care and health sciences, including experience in prehospital care. Initially, all transcripts were read repeatedly to gain a comprehensive understanding of the material. Meaning units, such as words, sentences or paragraphs, were identified in relation to the study’s aim, then condensed and abstracted into codes before being reformulated as items. This was further discussed among the co-authors to establish a solid foundation for the analysis. Grouping and comparing the condensed material led to the identification of the categories: knowledge, skills and attitudes (Erlingsson and Brysiewicz 2017). The constructed items subsequently underwent a content validation process guided by the recommendations described by Polit, Beck and Owen (Polit et al. 2007). Five researchers within the field of psychiatry and policing reviewed and rated the relevance of each item. An item content validity index (I-CVI) was calculated and guided decisions about revisions or rejections (19 items were revised and 1 rejected). The mean I-CVI for relevance was 0.98. The final questionnaire consisted of 57 items and was constructed using the online platform Survey & Reports, provided by Artologik. 

### Data collection and analysis

Data were collected in two rounds between September and October 2025, using a questionnaire sent by email. Initially, written consent and demographic data were collected, after which the experts were asked to rate the extent to which they agreed with each item, reflecting statements about competencies (knowledge, skills and attitudes) required of PPOs in encounters with individuals in suicidal crisis, using a five-point Likert scale, ranging from (1) ‘strongly disagree’ to (5) ‘strongly agree’. For analytical purposes, the scale was trichotomised to a three-point scale: 1–2 on the Likert scale represented “not agree”, 3 “neutral” and 4–5 “agree”. The consensus level was set at ≥ 80% prior to data collection. Hence, any statement for which ≥ 80% of the experts agreed was considered to have achieved consensus. Furthermore, the experts were invited to propose additional competencies via free-text responses. These were subsequently analysed, constructed into items, categorised and incorporated into Round Two alongside items that had not reached consensus in Round One. In Round Two, the experts were presented with their individual rating from Round One, along with the group’s mean rating for each item, to consider when responding. For each round, three reminders were sent by email within the first 12 days, and each questionnaire was open for 15 days. For data analysis, descriptive statistics (mean, standard deviation and range) were used.

### Ethical considerations

The study followed the principles of the Declaration of Helsinki (World Medical Association 2024). An advisory statement from the Swedish Ethical Review Authority (2023-07149-01) was obtained prior to the study. The experts received written information about the study, including a statement that participation was voluntary. They were assured of confidentiality and informed of their right to withdraw at any time without providing a reason. Written informed consent was obtained prior to completing the questionnaire in Round One.

## Results

Of the 50 experts invited, 47 responded to Round One (94%) and were subsequently sent a second questionnaire. In Round Two, 43 experts responded (91.5%), bringing the total response rate across both rounds to 86%. Initially, 57 items were included in the questionnaire, categorised into knowledge (*n* = 13), skills (*n* = 27), and attitudes (*n* = 17). An additional four were generated from free-text responses in Round One, bringing the total number of items to 61. Consensus was subsequently reached on 50 items based on expert ratings, distributed into the categories of knowledge (*n* = 14), skills (*n* = 22) and attitudes (*n* = 14) (Table 2).

**Table 2:** Consensus items categorised into Knowledge (K), Skills (S) and Attitudes (A)

Item No.	Item	Mean (M)	SD^1^	Round^2^	Range
Category – Knowledge					
K.1	Factors in police interaction that may exacerbate a suicidal crisis	4.8	0.43	1	4–5
K.2	The suicidal process	4.7	0.52	1	3–5
K.3	Legislation on compulsory psychiatric care	4.6	0.61	1	3–5
K.4	Police organisational resources available to provide assistance in suicidal crisis situations	4.5	0.68	1	2–5
K.5	The phenomenon of suicide by cop	4.5	0.68	1	2–5
K.6	Underlying factors that may contribute to a suicidal crisis	4.4	0.67	1	2–5
K.7	The impact of violence and coercion on mental health	4.4	0.64	1	3–5
K.8	Structure and role of mental health services, including emergency psychiatry and outpatient care	4.3	0.66	2	3–5
K.9	General pathology related to mental disorders	4.1	0.61	1	3–5
K.10	Substance misuse and its association with mental health disorders	4.1	0.61	2	1–5
K.11	Basic principles of pathology in relation to panic disorder	4.0	0.61	2	2–5
K.12	Basic principles of pathology in relation to depressive disorders	4.0	0.65	1	2–5
K.13	Basic principles of pathology in relation to psychotic disorders	4.0	0.69	1	2–5
K.14	The responsibilities of EMS and FRS during a suicidal crisis	4.0	0.79	1	2–5
Category – Skills					
S.1	Be persistent and patient during the encounter	4.8	0.43	1	4–5
S.2	Use active listening	4.8	0.47	1	3–5
S.3	Manage physical care needs (e.g., stop bleeding after a self-inflicted cut)	4.7	0.51	1	3–5
S.4	Collaborate effectively within a patrol	4.7	0.51	1	3–5
S.5	Adapt one’s approach based on the individual’s unique life circumstances during a suicidal crisis	4.7	0.52	2	3–5
S.6	Manage one’s own emotional responses during interactions	4.7	0.59	1	3–5
S.7	Use appropriate non-verbal communication (body language and facial expressions)	4.6	0.49	1	4–5
S.8	Use de-escalating communication	4.6	0.54	1	3–5
S.9	Read between the lines in verbal communication and identify what is relevant	4.6	0.54	1	3–5
S.10	Assess non-verbal communication (body language and facial expressions)	4.5	0.62	1	2–5
S.11	Prioritise information and situational cues in order to make well-considered decisions	4.5	0.65	1	2–5
S.12	Apply physical intervention techniques and tactical methods to manage threats or violence from an individual in suicidal crisis	4.4	0.52	2	3–5
S.13	Assess mental state in order to determine whether there is a need for care	4.4	0.61	1	2–5
S.14	Select strategies based on available resources (both police and healthcare)	4.4	0.68	1	3–5
S.15	Assess irrational behaviour	4.4	0.71	1	2–5
S.16	Redirect suicidal thinking through motivational communication	4.4	0.71	1	2–5
S.17	Adapt one’s approach according to the maturity level of the individual in suicidal crisis (children/adolescents versus adults/older persons)	4.4	0.71	1	2–5
S.18	Apply a low-arousal approach	4.4	0.79	1	1–5
S.19	Assess the risk to life and health in cases of bodily injury associated with a suicide attempt	4.3	0.66	1	3–5
S.20	Intervene with physical force during an ongoing suicide attempt	4.3	0.75	1	2–5
S.21	Manage relatives and bystanders who are present near the individual in suicidal crisis	4.2	0.71	1	2–5
S.22	Intervene with physical force in situations of imminent suicide risk	4.0	0.73	2	2–5
Category – Attitudes					
A.1	Non-judgemental	4.8	0.40	1	4–5
A.2	Demonstrates compassion	4.8	0.43	1	4–5
A.3	Conveys a sense of calm	4.8	0.47	1	3–5
A.4	Characterised by empathy	4.7	0.67	1	2–5
A.5	Considers the risk of violence towards oneself and colleagues	4.6	0.53	1	3–5
A.6	Demonstrates genuine presence	4.6	0.61	1	3–5
A.7	Demonstrates adaptability in interactions	4.5	0.58	1	3–5
A.8	Maintains composure in life-threatening situations	4.5	0.58	1	3–5
A.9	Demonstrates a problem-solving and flexible approach	4.4	0.77	1	2–5
A.10	Respects the dignity and privacy of individuals in suicidal crisis	4.3	0.77	1	2–5
A.11	Demonstrates the courage to engage with emotions in an open and honest manner	4.2	0.70	1	3–5
A.12	Values the need for further knowledge	4.2	0.79	1	2–5
A.13	Shows preparedness to respond when background details are scarce, including mental health or suicidal history	4.2	0.81	1	1–5
A.14	Demonstrates the ability to view the situation from the perspective of the person in suicidal crisis	4.1	0.80	1	2–5

## Standard deviation (SD). 2 Consensus reached in round

### Round 1

**Fig. 1:**
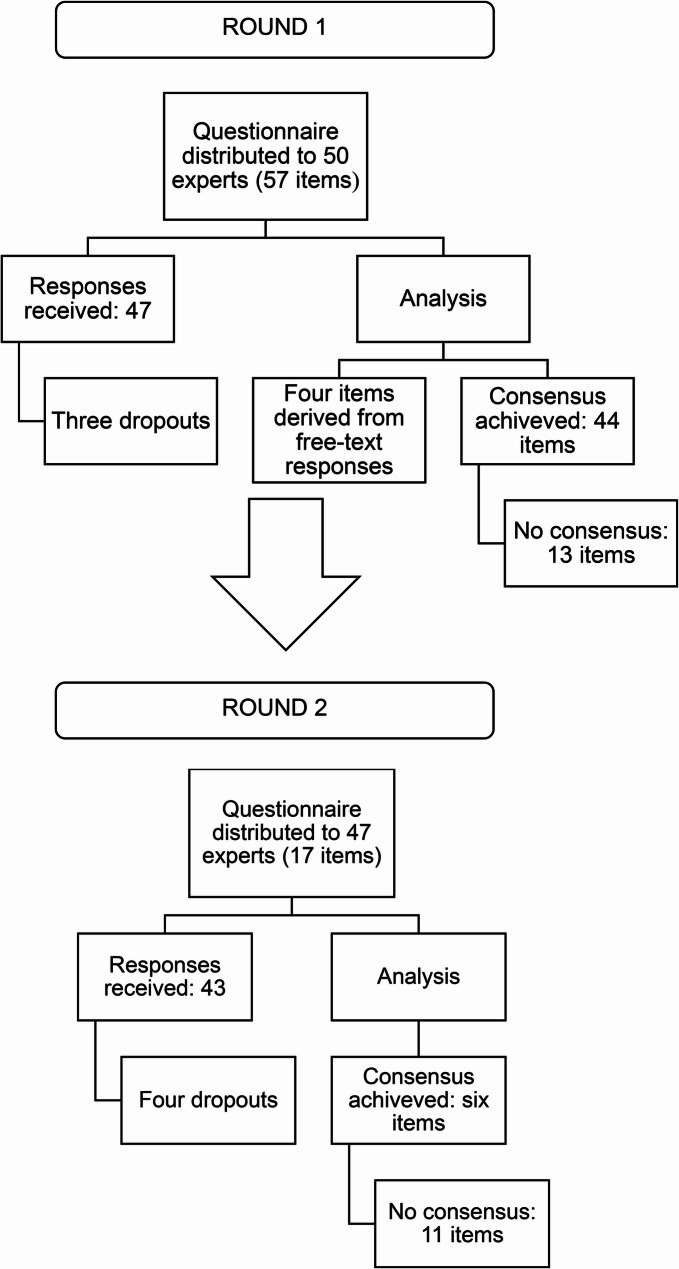
Delphi flowchart of the two rounds

A total of 44 items reached the consensus level of ≥ 80%, distributed across the categories of knowledge (*n* = 11), skills (*n* = 19) and attitudes (*n* = 14). An additional four items were generated from free-text response (see Fig. 1).

### Round 2

Of the 17 items, comprising both non-consensus items and those generated from free-text responses in Round One, six reached the consensus level of ≥ 80%, distributed across the categories of knowledge (*n* = 3), skills (*n* = 3), and attitudes (*n* = 0) (see Fig. 1).

## Discussion

The findings identify competencies categorised into knowledge (*n* = 14), skills (*n* = 22) and attitudes (*n* = 14). These competencies range from police-specific, such as tactical skills, to those more usually associated with mental health care, such as knowledge of pathology related to mental disorders. Acting under the legal mandates of compulsory legislation, as well as the ability to de-escalate situations and make rapid, informed decisions, was also identified as relevant competencies.

The interaction between PPOs and people in suicidal crisis is characterised by the necessity for officers to make legal decisions that affect personal liberty and should be conducted in a manner that avoids stigma, which can otherwise define these encounters (Kara 2014). The findings are discussed in relation to the principles of procedural justice theory (PJT), consisting of voice, trust and neutrality, and dignity and respect, as well as police education. References to numbered items in parentheses indicate their category: K = Knowledge, S = Skills, and A = Attitudes, as defined in the Results section. PJT concerns the fairness of decision-making processes and the policies guiding allocation decisions, which in turn contribute to the legitimacy of authority. Satisfaction with legal, or in a psychiatric context, clinical decisions is primarily influenced by the perceived quality of the procedure itself, rather than by the actual outcome. The subjective experience may have a significant impact on clinical outcomes; in other words, an individual’s perception of the process itself can shape the response to psychiatric treatment (Aluh et al. 2025, Tyler and Fagan 2008). Conversely, contact with the police may adversely affect mental health, particularly when such interactions are perceived as unjust (Kyprianides et al. 2025, Kyprianides and Bradford 2025). Recognising the potential negative impact of interactions with PPOs, particularly when characterised by violence and coercion, was identified as a relevant competency in this study (K.1, K.7).

### Voice

Procedural justice goes beyond ensuring compliance with legislation and involves therapeutic factors that can assist in the recovery process for people who experience mental ill-health (Kara 2014, Mazerolle et al. 2013). A key component of this is the opportunity to explain one’s views and behaviour before the police make decisions. This reflects the value of being listened to and the level of participation afforded in the decision-making processes (Aluh et al. 2025). Complementing this, previous research highlights the importance of transparent decision-making, whereby PPOs explain their actions and underlying rationale, communicate processes in a comprehensible manner, and outline subsequent steps. Such transparency may help to reduce uncertainty and address concerns in individuals experiencing suicidal crises (Lavoie et al. 2025). The results demonstrate a consensus regarding several competencies associated with de-escalation skills within the context of policing (S.1, S.2, S.6–S.10, S.16, S.18). These competencies align with crisis intervention training for law enforcement personnel (Davies and Dawson 2025). They emphasise key social skills, such as empathetic communication, active listening and the ability to adapt one’s approach to help individuals in crisis regain emotional control and engage in the decision-making process. For EMS personnel, recognising the individual’s lived experience was considered essential for building rapport and fostering collaborative engagement, although achieving this can be challenging during a suicidal crisis (Hammarbäck et al. 2024, Holmberg et al. 2025). This aligns with attitudes of respecting dignity, as well as the ability to view the situation from the perspective of the person in suicidal crisis (A.10, A.14). Irrational behaviour is common in cases of severe mental ill-health (Balfour et al. 2022). This may impede efforts to comprehend the individual’s perspective and level of participation in the decision-making processes. The ability to assess such behaviour remains crucial in order to preserve the individual’s voice and ensure they are heard, even though it can be challenging for both psychiatric professionals and PPOs (Lindridge et al. 2025, Morgan 2021). The experts considered this competency (S.15) important, as was the ability to assess the mental state in order to determine whether there is a need for care (S.13). They also emphasised knowledge of general pathology (K.9), the suicidal process (K.2) and its associated factors (K.6), as well as specific diagnoses such as substance misuse, psychosis, depression and panic disorder (K.10–K.13). As a PPO’s initial assessment may result in psychiatric care or community services, it inevitably carries significant implications for both compliance and the quality of such care. This underscores the need for collaboration between PPOs and healthcare professionals, including a handover that supports individual participation and seeks to ensure the client’s voice is heard (Morgan 2024). This study identifies a sound understanding of the healthcare system’s function, organisation and responsibilities, including those of EMS, as a core competency (K.8, K.11).

### Trust and neutrality

Neutrality in decision-making and the demonstration of trustworthiness are essential components of PJT, and thereby for fostering cooperation (Tyler and Fagan 2008). The results demonstrate that a relevant competence for PPOs is the ability to enforce legislation using physical force, if necessary, in encounters involving an imminent risk of suicide or and an ongoing suicide attempt (S.20, S.22). In such contexts, neutrality and respectful treatment, as far as practically achievable, and benevolence on the part of authority, are of importance (Galon and Wineman 2010, Boyle and Walsh 2021). Knowledge about compulsory legislation (K.3), the ability to make informed and well-considered decisions (S.11), maintaining composure under pressure (A.8), and adopting a non-judgemental attitude (A.1) were also identified as important competencies. However, such competencies do not necessarily lead to neutrality or the ability to demonstrate trustworthiness in their application. Structural and cultural features of policing, such as dismissive attitudes towards aspects of their profession that are aligned with social or mental health care, may undermine trustworthiness and compromise neutrality by fostering more authoritative and less empathetic interactions, with reduced attention to the individual’s perspective (Morgan 2024).

### Dignity and respect

Being met with dignity and respect is a fundamental component of PJT, and a core principle when engaging with people in vulnerable states (Tyler and Fagan 2008, Clua-García et al. 2021). A suicidal crisis entails profound vulnerability. While primarily linked to acute mental distress, it may also arise from socio-economic, ethnic or other marginalising factors, such as sexual minority status (i.e., minority stress) (Jadva et al. 2023). In the Nordic countries, ethnic minority youths reported mixed experiences when interacting with PPOs: they often felt protected and generally trusted the police, yet at the same time felt unjustifiably suspected, giving rise to strong perceptions of procedural injustice (Saarikkomäki et al. 2021). These encounters may require specific competencies, as PPOs operate from a position of legal authority and often engage with minorities in a vulnerable state of mind (Ramchand et al. 2022, Haller et al. 2020). The results indicate that the experts reached consensus on the importance of competencies such as showing empathy, demonstrating genuine presence, and engaging with compassion (A.2, A.4, A.6). However, the meaning of such competencies may vary depending on perspective and would benefit from further exploration from the perspective of people who have experienced suicidal crises (Shamsaei et al. 2020). These individuals often describe multidimensional suffering, including psychological pain as well as social, relational, and situational factors, highlighting the importance of incorporating experiential perspectives into such competencies (Shamsaei et al. 2020, Malekzadeh et al. 2025). Except for the consensus reached regarding the ability to adapt one’s responses according to developmental maturity (S.18), competencies that explicitly address dimensions of minority stress were not specifically identified. In this regard, it is important to consider how routine police encounters perceived as lacking dignity and respect—even when unrelated to suicidal crises—can have significant effects on the mental health of those involved, and later help-seeking behaviour. Evidence from the United Kingdom links such experiences to higher rates of self-harm and increased odds of suicide attempts among affected youths (Jackson et al. 2021). Prior research has similarly underscored the role of relational competencies, including the expression of genuine concern for the individual’s welfare and an empathetic and validating approach. Furthermore, humanising the interaction by promoting dignity, preserving self-worth, and fostering rapport has been described as key competencies in effective police encounters (Lavoie et al. 2025).

### Police education

The Nordic police forces have common characteristics and, in general, are considered to have a culture aligned with the principles of PJT (Ugelvik 2016, Damme et al. 2015, Laird and Charman 2023). This may stem from cultural, legal and operational elements, but it can also be institutionalised through education (Laird and Charman 2023, Skogan et al. 2015). Abderhalden and Alward (2024) found that a higher perception of procedural justice among prison inmates was associated with a reduced frequency of suicidal ideation. Hence, it carries important implications for the training of correctional staff (Abderhalden and Alward 2024). Sustaining procedural justice in policing relies on targeted efforts, with professional education occupying a central role in this process (Skogan et al. 2015, Cohen and Headley 2024). Selecting strategies based on available resources (S.14) was emphasised as an operational decision-making competency, highlighting the need for grounding in everyday policing. Although, in Sweden, it is uncommon and poorly quantified, the use of force remains part of policing, and cases where suicidal intent intersects with police use of firearms, sometimes referred to as ‘suicide by cop’, illustrate the ethical and operational complexity officers may face (Polismyndigheten 2024). Skills in utilising physical intervention techniques to manage threats or violence were considered important (S.12). The ability to evaluate one’s own need for further knowledge (A.12), indicating a requirement for reflective ability and self-awareness in relation to the subject matter, was identified as an important competency. Adaptability and a problem-solving orientation were also regarded as fundamental attitudes (A.7, A.9), even when operating under conditions of limited information and inadequate grounds for decision-making (A.13). To support the development of these competencies, reflective practice based on simulation-based learning that requires acting in accordance with compulsory legislation can be employed (Lavoie et al. 2022, Rantatalo and Karp 2016). Furthermore, integrating both the perspectives of the lived experience of being in a suicidal crisis and the expertise of psychiatric professionals, who foster an empathetic approach by emphasising therapeutic relationships, may enhance the quality of engagement in education (Kyprianides and Bradford 2025, Rantatalo and Karp 2016, Rask et al. 2025). Simulations can range from low-fidelity exercises, such as role-playing scenarios, to high-fidelity simulations, including immersive virtual reality training and trainer- or AI-driven educational avatars that interact dynamically with learners (Fink 2024, Muñoz et al. 2024, Lavoie et al. 2023).

### Limitations

This study used a modified Delphi technique with the ambition to generate items and, through a panel of experts, reach a collective viewpoint via a rigorous research process described in detail, aiming for high methodological rigour. Descriptions derived from semi-structured interviews with PPOs provided a grounding in frontline policing, although the reliance on interview data may have constrained the breadth of competencies compared to a broader literature base. The qualitive analysis, which formed the basis for developing the questionnaire, followed the method outlined by Erlingsson and Brysiewicz (2017). Trustworthiness was enhanced through collaborative discussions and refinement of the analysis among the co-authors. The items were subsequently subjected to content validation by five researchers using a CVI, thereby contributing to content validity (Polit et al. 2007). A limitation of a Delphi study lies in the somewhat ambiguous and inconsistent definition of an ‘expert’, which may refer to a specialist in the field, an informed individual, or someone possessing relevant knowledge, experience and expertise (Keeney et al. 2011, Keeney et al. 2006). In the present study, the definition of ‘expert’ was aligned with the aim of capturing competencies of relevance to policing in relation to encountering people in suicidal crisis. However, in the context of Delphi studies within mental health, the term ‘expert’ is more commonly associated with participants possessing formal psychiatric training, i.e. psychiatric professionals (Jorm 2015). An alternative definition of ‘expert’ might have produced different results. As the initial item generation was based on PPOs’ perspectives and the expert panel was aligned with policing practice, the study primarily reflects perspectives rooted in policing contexts. Hence, a limitation of this study is that competencies relevant from the perspective of people experiencing suicidal crisis, as well as those from mental health perspectives, are not fully represented. There is little agreement about the size of the expert group (Keeney et al. 2001). Given the definition of experts, a group of 50 was considered appropriate with the aim of providing representative information. As noted by Hansson et al. (2000), the size of the group can range from fewer than 15 to upwards of 60 experts. A challenge inherent to the iterative process in Delphi studies is the potential for expert attrition, particularly when using a large expert panel, which may contribute to validity concerns (Hasson et al. 2000). Fatigue can occur after as few as two rounds (Keeney et al. 2006). Using a modified Delphi approach in which the questionnaire was constructed prior to distribution to the expert panel, and limiting the process to two rounds, may have reduced this risk. The response rate of 86% indicates a satisfactory level of participant engagement, particularly considering the size of the group, thereby strengthening the validity of the results. The iterative revision of judgements, informed by the feedback mechanism in Round Two, strengthens internal validity, although additional rounds could potentially have further reinforced this. There is no universal agreement on the cut-off level for consensus in Delphi studies (Keeney et al. 2011). However, a ≥ 75% agreement level is frequently suggested as a minimal threshold for consensus (Keeney et al. 2006, Barrios et al. 2021). In the present study, a consensus level of ≥ 80% was chosen to ensure that the findings would be sufficiently robust, considering the transferability of the results into an educational context. External validity is restricted by cross-national differences in legislation governing compulsory interventions, organisational arrangements within policing (such as levels of centralisation and collaboration with healthcare services), and varying cultural attitudes towards mental health and suicide (Roché and Fleming 2022). However, the relative similarities in legislation, policing structures, and welfare systems across the Nordic countries may support a degree of transferability within this context (Laird and Charman 2023, Roché and Fleming 2022).

## Conclusion and implications

The results provide insight into the broad range of competencies required for PPOs, including policing-specific skills related to managing threats or violence, social skills for interpersonal communication, and psychiatric knowledge, such as understanding mental disorders. Additionally, the results underscore the importance of attitudinal competencies, such as engaging with compassion, adaptability, and a problem-solving orientation. Based on these conclusions, several implications emerge, as well as recommendations for future research.

- At the individual level, the results can offer guidance on competency requirements and help identify areas for professional development. This also requires PPOs to recognise the critical role they play and the breadth of competencies expected of them.
- At the group level, the results offer guidance for the development of curricula and educational initiatives. Education for PPOs should address the challenges of policing, including the mandate to act in accordance with compulsory legislation. Their ability to gain cooperation and the consequences of exercising authority should be reflected. Given the shared responsibility and collaboration among professionals on site, this highlights the importance of interprofessional learning with mental health professionals and EMS personnel.
- At an organisational level, the police response to people in suicidal crisis should be further developed. Given the broad range of competencies identified, many of which involve psychiatric knowledge and social skills associated with managing people in crisis, this implies strengthening co-response programmes and expanding psychiatric care available in out-of-hospital settings.

Further research should explore the development and implications of educational interventions and further investigate the nature of encounters between PPOs and people in suicidal crisis.

## Data Availability

The datasets generated and analyzed during are not publicly available due to confidentiality and ethical restrictions but are available from the corresponding author on reasonable request.
